# Muscle Antioxidant Enzymes Activity and Gene Expression Are Altered by Diet-Induced Increase in Muscle Essential Fatty Acid (α-linolenic acid) Concentration in Sheep Used as a Model

**DOI:** 10.3390/nu11040723

**Published:** 2019-03-28

**Authors:** Eric N. Ponnampalam, Vahid Vahedi, Khageswor Giri, Paul Lewandowski, Joe L. Jacobs, Frank R. Dunshea

**Affiliations:** 1 Animal Production Sciences, Agriculture Victoria Research, Department of Jobs, Precincts and Regions, Bundoora VIC 3083, Australia; khageswor.giri@ecodev.vic.gov.au; 2 Department of Animal Science, Moghan College of Agriculture and Natural Resources, University of Mohaghegh Ardabili, Ardabil 56971-94781, Iran; vahediv@uma.ac.ir; 3 Pelican Biological, Wallington VIC 3222, Australia; pelicanbiological@gmail.com; 4 Animal Production Sciences, Agriculture Victoria Research, Department of Jobs, Precincts and Regions, Ellinbank VIC 3821, Australia; Joe.Jacobs@ecodev.vic.gov.au; 5 Faculty of Veterinary and Agricultural Sciences, The University of Melbourne, Parkville VIC 3010, Australia; fdunshea@unimelb.edu.au

**Keywords:** dietary intervention, omega-3 fatty acid, linolenic acid, antioxidant enzyme activity, gene expression, performance, health and wellbeing, sheep

## Abstract

This study investigated the effect of dietary manipulations on muscle fatty acid composition, the activities and relative mRNA expressions of antioxidant enzymes and the relationship between muscle enzyme activity or mRNA expression and alpha linolenic acid (ALA) concentration in sheep. Eighty-four lambs blocked on liveweight were randomly allocated to four dietary treatments, lucerne pasture (Lucerne), annual ryegrass pasture (Ryegrass), feedlot pellets (Feedlot) or annual ryegrass plus feedlot pellets (RyeFeedlot). After six weeks of feeding, lambs were slaughtered and within 30 min post-mortem, samples collected from the *longissimus lumborum* (LL) muscle for RNA isolation and measurement of antioxidant enzyme activities. At 24 h post-mortem, LL samples were collected for determination of fatty acid concentrations. Feedlot treatment decreased ALA, eicosapentaenoic (EPA), docosapentaenoic (DPA) and docosahexaenoic (DHA) concentrations compared with other treatments and increased linoleic acid (LA) and arachidonic acid (AA) compared with Lucerne and Ryegrass (*p* < 0.001). The activity of Glutathione peroxidase (GPX1, *p* < 0.001) and Superoxide dismutase (SOD2, *p* < 0.001) enzymes in the muscle increased with Lucerne compared to other treatments. Lucerne increased muscle *gpx1* mRNA expression by 1.74-fold (*p* = 0.01) and 1.68-fold (*p* = 0.05) compared with Feedlot and other diets, respectively. The GPX1 (*r*^2^ = 0.319, *p* = 0.002) and SOD2 (*r*^2^ = 0.244, *p* = 0.009) enzyme activities were positively related to ALA. There was a positive linear relationship between muscle *gpx1* (*r*^2^ = 0.102, *p* = 0.017) or *sod2* (*r*^2^ = 0.049, *p* = 0.09) mRNA expressions and ALA concentration. This study demonstrates that diet can affect concentrations of ALA and other fatty acids as well as change activities and gene expression of antioxidant enzymes in muscle. Increased antioxidant activity may, in turn, have beneficial effects on the performance, health and wellbeing of animals and humans.

## 1. Introduction

Dietary fat concentration and composition are two of the major factors influencing basal energy metabolism, body fat deposition and muscle fatty acid composition [1,2,3,4]. For example, humans and animals consuming diets high in grains and oilseeds/meals such as corn, sunflower, peanut often deposit high concentrations of n-6 fatty acids, mainly linoleic acid (LA), the precursor n-6 fatty acid and their derivatives in body tissues [5]. Conversely, animals and humans consuming greater amounts of green plant materials and feeds from marine origin deposit greater concentrations of n-3, mainly alpha linolenic acid (ALA), the precursor n-3 fatty acid and their derivatives [1,6,7,8]. These effects were not only due to diets being rich in n-3 and n-6 precursor fatty acids but also due to competition between these fatty acids for absorption at the enterocyte and peripheral tissue level, as they share common enzymes for desaturation and elongation processes [3,9,10]. Dietary intervention studies performed in human and laboratory animals with supplementation of n-3 fatty acids revealed beneficial outcomes on brain development, vision improvement as well as health and wellbeing aspects [11,12,13,14]. This has heightened the interest in health-enhancing food products, which has led to producers and retailers focusing on farm animal products such as meat and milk with increased concentrations of health-promoting nutrients such as essential fatty acids, minerals and antioxidants [15,16,17,18].

It is also important to maintain the antioxidant status of tissue systems through dietary intervention to avoid the oxidation of essential polyunsaturated fatty acids (n-3 and n-6) in muscles and subsequently ensure no off-flavour development of food prior to consumption [19,20]. This latter strategy is only necessary if the antioxidant status of the muscle and other tissues are deficient i.e., below the threshold at the time of animal exsanguination in order, to avoid or slow down the oxidation process in muscle foods post-mortem [21]. Oxidative stress in living organisms results from an imbalance between the production of reactive oxygen species (ROS) and the antioxidant defence, which can cause lipid, protein and DNA damage in cells and tissues, resulting in the development of certain diseases and pathophysiological changes [22,23,24]. To protect the cells and tissues from oxidative damage, living organisms maintain a multi-dynamic antioxidant defence system. This system contains non-enzymatic antioxidants (such as vitamins, carotenoids, polyphenols, flavonoids and proteins containing thiol groups,) and enzymatic antioxidants such as glutathione peroxidase (GPX), superoxide dismutase (SOD) and catalase [21,25]. The general belief is that these enzymatic and non-enzymatic antioxidants present in the organelle (circulatory and tissue) systems have the capacity to break down free radical formations by directly scavenging the free radical forming agents such as H_2_O_2_ or chelate the metal ions serving as an oxidative base such as Fe^2+^, which in turn minimise the development of reactive oxygen species. The latter process indeed avoids lipid, protein and DNA damage in the cellular systems, improves immune responses and protect animals (organisms) from oxidative stress and disease development [22,26].

It has been well established from biological, biochemical and behavioural studies that the consumption of diets high in omega-3 fatty acids has many health advantages such as reducing the risk of heart disease, inflammation, obesity, cancer, hypertension, depression and asthma [14,27]. These effects can be mediated through autoimmune functions, pathological responses, insulin-stimulated energy disposal, gene expressed signal transduction, eicosanoid metabolism. If omega-3 fatty acids are beneficial in improving the health and wellbeing of humans and animals, it could possibly be through amelioration of oxidative stress, immune suppression and cell damage enhanced by the antioxidant defence. In this case, there should be associative effects between activity and/or relative mRNA expressions of antioxidant enzymes and diet-induced changes in muscle essential n-3 fatty acids, for example, ALA. Feeding alfalfa (lucerne) forage or supplemental flaxseed to sheep increased muscle essential n-3 fatty acid concentration, mainly ALA, in sheep [8,28]. Therefore, the hypothesis to be tested in the present experiment is that if there was a significant increase in muscle ALA concentration with dietary intervention, it should alter the activity and(or) relative mRNA expression of antioxidant enzymes through cellular biochemical mechanisms and signal transductions. To test this, we used feedlot diets that generally provide high proportions of LA so that muscle n-6 levels will be increased significantly to compare with a pasture diet such as lucerne (alfalfa) that may enrich muscle n-3 levels, providing an extreme range of fatty acids for comparisons.

## 2. Materials and Methods

### 2.1. Experimental Design, Animal Numbers and Dietary Treatments

Animal feeding experimentation was conducted at the Rutherglen research facility, Victorian Government (Department of Jobs, Precincts and Regions; DJPR) after approval was obtained from the DJPR Agricultural Research and Extension Animal Ethics Committee (AEC#: 2012-19). All procedures were conducted in accordance with the Australian Code of Practice for the Care and Use of Animals for Scientific Purposes [29].

Second cross lambs born to Poll Dorset sires and Border Leicester Merino dams were used in the experiment. The lambs were born in July 2012 and raised on annual ryegrass (*Lolium rigidum* L.) pasture with their dams until weaning at ~10-week-old age. Eighty-four male wether (*n* = 42) and female ewe (*n* = 42) lambs were used in this experiment. After weaning, the lambs were separated from their dams and allocated to their four finishing diets in separate paddocks. Lambs were blocked on liveweight and then within each block of four lambs randomly allocated to treatments. The four finishing diets were: standard commercial feedlot pellets (*n* = 24, Feedlot); lucerne (*Medicago sativa* L.) pasture (*n* = 24, Lucerne); annual ryegrass with sub clover (*Trifolium subterraneum* L.) pasture (*n* = 18, Ryegrass) or annual ryegrass pasture plus feedlot pellets (500 g/day/head) (*n* = 18, RyeFeedlot). There were four replicates of Feedlot and Lucerne paddocks each having six animals per replicate and three replicates of Ryegrass and RyeFeedlot paddocks each having six animals per replicate for a total of 84 lambs.

The feeding of lambs, pasture allocation and animal performance have been reported in detail elsewhere [30]. In brief, lambs were adapted to their experimental diets for 2 weeks (0–2 week) and then fed their full treatment diets for the following six weeks (2–8 week). The Ryegrass and RyeFeedlot treatments replicates were implemented in the same pasture area thus allowing the lambs to graze annual ryegrass sward of similar nutritive characteristics within the forage component of the two diets. Lambs fed their Lucerne, Ryegrass and RyeFeedlot diets were moved to new paddocks (new pasture allocation) every week whereas the Feedlot groups were maintained at their particular locations throughout the feeding. Liveweight of animals were recorded weekly. All lambs had acceptable body weight gains ranging from 200–300 g/day, which can be categorised as moderate to high growth under standard lamb production systems. The nutritional composition of diets has been previously presented [30] and, also presented here (Table 1). The Ryegrass treatment had a low crude protein (CP) concentration compared to other treatments. This was a result of the annual ryegrass having already entered its reproductive phase resulting in a decline in CP concentration. This has resulted in different performance and carcass weights between dietary treatments and this has been reported in a previous [30] publication.

### 2.2. Animal Slaughter, Muscle Sample Collection and Fatty Acid Analysis

At the completion of the finishing period, lambs were transported to a commercial abattoir in Kyneton, Victoria and slaughtered after fasting for 18 h. At 30 min post-slaughter, a 2 g muscle *longissimus lumborum* (LL) sample was collected from the same muscle location in the body and immediately snap frozen in liquid nitrogen before storing at −80 °C for the analyses of mRNA expression and activity of antioxidant enzymes. At 24 h post-mortem, carcass weights, carcass fatness and the ultimate pH of the muscle LL was recorded as a standard commercial routine procedure. Muscle LL from the hindquarter over the lumbar region was removed from the left side of the carcass and duplicate samples (~25 g) were collected and stored at −20 °C for the determination of fatty acids. One set of samples from all animals was freeze-dried, homogenised and approximately 0.5 g was used for fatty acid extraction using a modified procedure as reported earlier [31].

### 2.3. Determination of Antioxidant Enzyme Activity

The frozen samples allocated were ground and antioxidant enzymes activity was determined enzymatically according to the methods reported by others [32]. The activity of the GPX enzyme was determined at 340 nm using a commercial assay kit (Cayman chemical, Redfern, NSW, Australia) in which oxidation of NADPH was monitored spectrophotometrically, whereas reductase activity was determined using H_2_O_2_ as a substrate. The activity of SOD enzyme was determined using a commercial assay kit (Cayman chemical, Redfern, NSW, Australia) by measuring the production of superoxide radicals at 440 nm, where one unit of activity was defined as the amount of enzyme needed to exhibit 50% dismutation of the superoxide radical. Total protein concentration in muscle LL tissue was determined with a bicinchoninic acid protein assay (Sigma Aldrich, Castle Hill, NSW, Australia). The activity of GPX was expressed as nmol/min/mg protein in muscle tissue whereas SOD was expressed as U/mg protein in muscle tissue.

### 2.4. Gene Expression Analyses

#### 2.4.1. Sample Preparation, RNA Isolation and cDNA Synthesis

Muscle LL samples were subjected to extraction and evaluation of gene expression. Isolation of total RNA from muscle was performed using Trizol Isolation Reagent (Life Technologies Australia Pty Ltd., Mulgrave, Australia) and PureLink RNA Mini Kit (Ambion, Life Technologies Australia Pty Ltd., Mulgrave, Australia) following the protocol recommended by the manufacturer. The extracted RNA was then diluted with 50 µL of RNase free water and stored at −80 ℃ until analysis performed.

The quality and quantity of the RNA extracted were evaluated by the Experion System Automated Electrophoresis Station (Bio-Rad Laboratories Inc., Gladesville, NSW, Australia) with the Experion StdSens Analysis Kit (Bio- Rad Laboratories Inc., Gladesville, NSW, Australia). For determination of RNA concentration and the ratio of 28S to 18S, the electropherograms were analysed for each sample. All the samples had an RNA quality indicator (RQI) higher than 9.0. Total RNA (8 μL) was then reverse transcribed into cDNA using Superscript™ III First-Strand Synthesis System Reagents (Invitrogen Life Technologies Pty Ltd., Mulgrave, Vic, Australia, #18080051) for reverse transcription polymerase chain reaction (RT-PCR) according to the protocol recommended by the manufacturer. Random hexamers were used as the primer during cDNA synthesis.

#### 2.4.2. Real-Time Quantitative PCR

The relative abundance of mRNA for each gene of interest was determined using real-time quantitative PCR (RT-qPCR) techniques. The genes evaluated included Carnitine palmitoyl transferase I-β (*cpt1β*), Glutathione peroxidase 1 (*gpx1*), Glutathione peroxidase 4 (*gpx4*), Nuclear factor kappa B (*nfκB*), Superoxide dismutase 1 (*sod1*), Superoxide dismutase 2 (*sod2*), Uncoupling protein 2 (*ucp2*). The custom forward and reverse primers for the gene of interest were designed using Invitrogen’s OligoPerfect Designer on the basis of ovine nucleotide sequences obtained from the National Center for Biotechnology Information (NCBI) nucleotide database and synthesized by GeneWorks (Thebarton, SA, Australia). Primer sets for the genes are shown in Table 2.

The PCR quantification was performed in 25-μL reactions (including 1 to 3 μL of forward and reverses primer, depending on optimized concentration, and 2 μL of sample cDNA) prepared according to the manufacturer’s instructions using SYBR GreenER Supermix (Life Technologies Pty Ltd., Mulgrave, Vic, Australia), and SYBR green fluorescence was quantified in triplicate with the iQ5 Single Colour Real Time PCR Detection System (BioRad Laboratories Inc., Gladesville, NSW, Australia). Each assay plate contained nontemplate control and a positive control (pooled cDNA), and a standard curve (five serial dilutions of a pooled cDNA sample) was run for each gene to determine the amplification efficiency of the respective primer pair.

The thermocycling conditions carried out for each assay run for all genes were initial denaturation at 95 °C for 3 min followed by 40 cycles of 95 °C for 10 s and 52 °C to 60 °C for 45 s (annealing temperature optimised depending on primers) and 72 °C for 1 min. Optical detection was performed at 72 °C. After amplification, a melt curve (95 °C for 1 min, 55 °C for 1 min, and then 40 cycles of 55 °C for 10 s) was included in the protocol to validate primer specificity and amplification of a single PCR product.

Analyses of amplification plots were performed with the iQ5 Optical System Software version 2.0 (Bio-Rad Laboratories Inc., Gladesville, NSW, Australia). All samples were normalised to the housekeeper gene (β-actin), which was used as the loading control. In other words, the genes of interest were loaded with a housekeeping gene in 96-well plates. Relative gene expression was analysed using the comparative delta CT method [33].

### 2.5. Statistical Analyses

Data were analysed using ANOVA procedure in GenStat 17th edition (VSN International, Hertfordshire HP2 4TP, UK) [34] with treatment used as the main effect and paddock as the block structure in order to obtain the predicted means for muscle individual fatty acid composition, mRNA expression and activities of antioxidant enzymes. *F*-tests were used to determine the overall significant difference among the predicted means, whereas the difference between two predicted means was judged to be significant if it was at least two times the average standard error of difference (SED). Relationships of muscle LL glutathione peroxidase (GPX1), superoxide dismutase (SOD2) enzyme activities, mRNA expression of *gpx1* (∆CT) and *sod2* (∆CT) with individual n-3 and n-6 fatty acids were analysed using a REML mixed model in GenStat 17th edition. In these REML analyses, the *a priori* effect of paddocks within a treatment was included as a random effect to account for any variability due to paddocks within a treatment. Plots of residuals versus fitted values were examined to check for any extreme outliers and to judge the necessity of data transformation to satisfy the assumption of normality with constant variance.

## 3. Results

### 3.1. Influence of Diet on Muscle Fatty Acid Composition

There was a clear indication of diet-induced variation in muscle n-3 (ALA, EPA, DPA, DHA) and n-6 (LA, AA) fatty acid deposition. Feedlot fed lambs had lower (*p* < 0.001) concentrations of muscle ALA, EPA, DPA and DHA compared with other dietary treatments. Muscle concentrations of LA and AA were lower (*p* < 0.001) for Lucerne- and Ryegrass-fed lambs compared with Feedlot-fed lambs, whereas Ryefeedlot treatment lambs had intermediate levels. Dietary treatments did not affect muscle total fat concentration or major fatty acid groups such as saturated (C14:0, C16:0, C18:0), and monounsaturated (C16:1, C18:1) fatty acids (Table 3).

### 3.2. Influence of Diet on Muscle Antioxidant Enzyme Activity and Gene Expression

**Figure 1:**
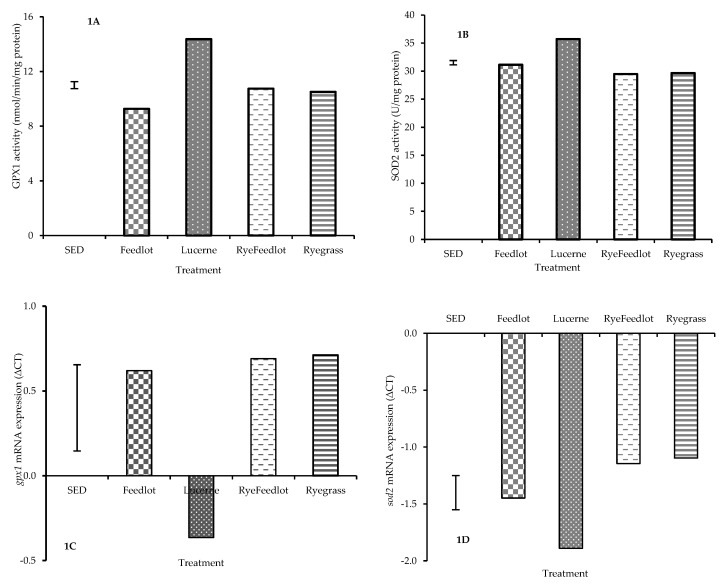
Dietary effects on muscle glutathione peroxidase 1 (GPX1) (**A**) and superoxide dismutase 2 (SOD2) (**B**) enzyme activities, and *gpx1* (**C**) and *sod2* (**D**) mRNA expressions.

Muscle antioxidant enzyme activities were higher in Lucerne-fed lambs compared with all other treatments. Activity of GPX1 (*p* < 0.001) and SOD2 (*p* < 0.001) enzymes in the LL muscle were increased by the Lucerne diet compared to other dietary treatments (Figure 1A,B). There were no significant effects of different feeding systems (*p* > 0.05) on muscle *cpt1β, gpx4, nfkB, sod1* and *ucp2* mRNA expression. Gene expression of GPX1 and SOD2 enzymes were greatest (i.e., lowest delta CT) for Lucerne among all treatment groups (Figure 1C,D). The Lucerne diet increased muscle *gpx1* mRNA expression by 1.74-fold (*p* = 0.01) and 1.68-fold (*p* = 0.05) compared with Feedlot and other treatments, respectively. Relative to Feedlot, muscle LL *sod2* mRNA abundance was increased by 1.98-fold (*p* = 0.05) with Lucerne but did not differ from Ryegrass or Ryefeedlot (*p* > 0.05) diets (Figure 1C,D).

### 3.3. Relationship between Muscle Essential Fatty Acids (n-3 and n-6 PUFA) and Antioxidant Enzyme Activity or Gene Expression

**Figure 2:**
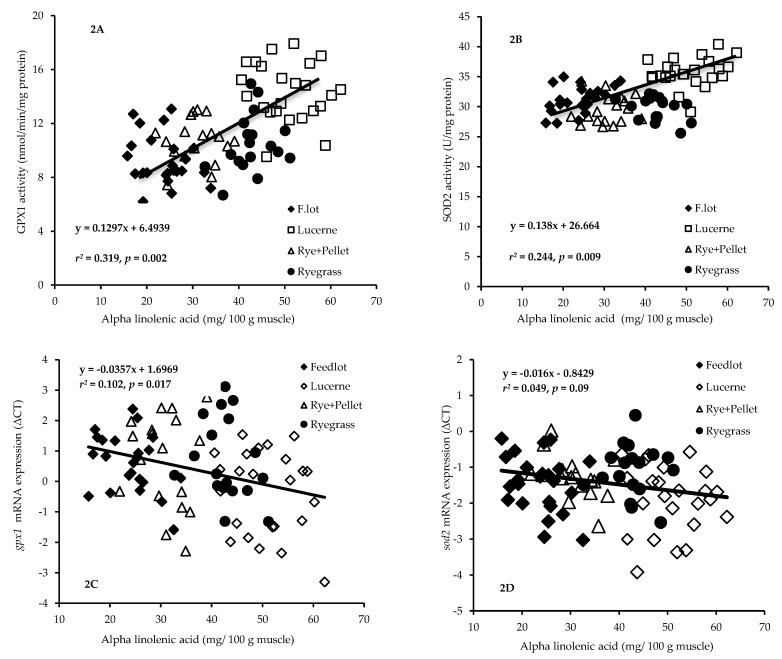
Relationship between muscle glutathione peroxidase 1 (GPX1) (**A**) or superoxide dismutase 2 (SOD2) (**B**) enzyme activity and alpha linolenic acid (ALA) concentration. Muscle GPX1 (*r*^2^ = 0.319, *p* = 0.002; Figure 2A) and SOD2 (*r*^2^ = 0.244, *p* = 0.009; Figure 2B) enzyme activities were linearly related to ALA (C18:3n-3). In Figure 2A,B, F.lot = Feedlot and Rye+Pellet = RyeFeedlot. Relationship between muscle *gpx1* (**C**) or *sod2* (**D**) mRNA expression (Delta CT) and ALA. A positive (*r*^2^ = 0.102, *p* = 0.017, Figure 2C) linear relationship of mRNA expressions of *gpx1* with ALA was observed whereas the mRNA expressions of *sod2* tended (*r*^2^ = 0.049, *p* = 0.09, Figure 2D) to be related to ALA concentration. In Figure 2C,D, Rye+Pellet = RyeFeedlot. In Figure 2A–D, each point represents an individual animal.

The REML mixed model analyses documented significant positive linear relationship between GPX1 enzyme activity and ALA (*r*^2^ = 0.319, *p* = 0.002; Figure 2A) and EPA (*r*^2^ = 0.218, *p* = 0.022; Figure not shown) concentrations but not with any other n-3 and n-6 fatty acids. Muscle SOD2 enzyme activity was positively (*r*^2^ = 0.244, *p* = 0.009) linearly related to ALA (Figure 2B). However, SOD2 enzyme activity was not significantly related to any other n-3 and n-6 fatty acids. We observed a significant positive (*r*^2^ = 0.102, *p* = 0.017) linear relationship between mRNA expressions of *gpx1* (lower delta CT) and ALA (Figure 2C). None of the other n-3 and n-6 fatty acids were significantly related to *gpx1* mRNA expression. The mRNA expressions of *sod2* tended (*r*^2^ = 0.049, *p* = 0.09) to be related to ALA concentration (Figure 2D) but not to any other n-3 and n-6 fatty acids.

## 4. Discussion

We report for the first time that diet-induced increases in muscle ALA concentration upregulate the activity and gene expression of GPX1 and SOD2 antioxidant enzymes, believed to be through altered cellular biochemical actions and signal transduction pathways (Table 4). The upregulation was shown by a positive relationship between antioxidant enzyme gene expression of *gpx1* and ALA or *sod2* and ALA (i.e., a lower delta CT is associated with upregulation of a gene), and a positive relationship between antioxidant enzyme activity of GPX1 and ALA or SOD2 and ALA in muscle LL tissue. We also observed that ALA deposition in muscle LL was influenced by the diet that animals were being fed. We have previously shown that these essential omega-3 fatty acids were stored in the muscle membranes as phospholipid bilayer [7], which in turn can further involve in altering muscle membrane nutrient permeability and cellular signalling actions [3,11]. We state that this may have potential benefits to performance and health and wellbeing of animals and humans as antioxidant enzymes can alleviate the impact of oxidative stress, impaired immune function and metabolic disorders associated with nutritional deficiencies, extreme heat/cold or other stress-induced events such as metabolic syndrome.

### 4.1. Diet and Muscle Fatty Acid Composition

It is well known that humans and animals cannot synthesise essential parent (precursor) fatty acids of LA and ALA *de novo* and therefore these fatty acids must come from the diet. These parent fatty acids are the source for the formation of their products of long-chain n-3 fatty acids of eicosapentaenoic (EPA, C20:5), docosapentaenoic (DPA, C22:5) and docosahexaenoic (DHA, C22:6) as well as n-6 fatty acid of arachidonic (AA, C20:4) acids, through desaturation and elongation processes, known to offer many health benefits and important for the growth and development of individuals [9,10,14,27]. It was interesting to note that Feedlot feeding reduced ALA concentration by 50% in muscle compared with Lucerne whereas Ryegrass and RyeFeedlot groups had 15% and 40% lower than Lucerne. This, in turn, resulted in 25% reduction in all long-chain n-3 fatty acids of EPA+DPA+DHA in Feedlot animals when compared with those fed Lucerne, although the other two feeding strategies delivered the same amounts as Lucerne fed animals. Linoleic acid concentration was increased by 40%–45% in Feedlot animals compared to other treatments. These findings indicate that dietary treatments clearly affected LA and ALA concentrations in muscle tissue and when Feedlot was combined with Ryegrass (RyeFeedlot) there was a partial reduction in ALA and complete reduction in LA due to the competition between the parent n-3 and n-6 fatty acids for elongation and desaturation process [3,9,27]. It was also noted that although significant, the products of long-chain n-3 (EPA, DPA, DHA) and n-6 (AA) were small in magnitude compared to their parent ALA and LA indicating that the efficiency of conversion was low as reported by many others [7,8,11,12,17] as opposed to feeding diets rich in long-chain n-3 and n-6 fatty acids directly. A previous study conducted by our group showed that flax meal at 10% or algae at 2% of diets fed to sheep significantly increased ALA or DHA, respectively [35]. As there were no significant dietary effects in the major saturated fatty acids of C14:0, C16:0 and C18:0 as well as major monounsaturated fatty acids of C14:1, C16:1 and C18:1, these results will not be discussed. The non-significant differences cannot be explained easily but the shorter feeding length (6 weeks) used in this study may in part be a contributing factor.

### 4.2. Gene Expression and Activity of Enzymes Associated with Dietary Intervention

The expression and activity of enzymes can be regulated by vitamins, minerals and fatty acids from diets and the hormone levels circulating within biological systems. To the best of our knowledge, no information is available on dietary intervention influencing muscle PUFA concentration and resultant altered activity and gene expression of GPX1 and SOD2 antioxidant enzymes in muscle tissues of sheep or humans. There have been several studies conducted in humans, rats, chicken and cattle investigating the effect of dietary PUFA on mRNA abundance of lipogenic enzymes of carnitine palmitoyl transferase 1 (CPT-1), acetyl-CoA carboxylase (ACC), stearoyl-CoA desaturase (SCD), delta-6 (∆-6) desaturase, delta-5 (∆-5) desaturase, fatty acid synthase in relation to fat metabolism (deposition, energy expenditure, oxidation), obesity, insulin resistance and other autoimmune responses [1,8,11,36,37,38,39]. It was summarised [39] that dietary manipulation of fatty acid composition in ruminants is mediated, at least partially, through the regulation of lipogenic enzymes expression; and that the regulation is tissue specific as observed in longissimus muscle and subcutaneous adipose tissue of bulls. It was found that the mRNA abundance of *fads1*, *fads2*, *acox1* and *cpt1* were not affected in breast muscle of chickens when flax oil (2.5% linseed oil in the diet) rich in ALA was fed to chickens [38] compared with those fed a control diet having 4% soybean oil. In the meantime, when grass rich in ALA was fed to cattle [36], the mRNA abundance of *fads1*, *fads2*, *acox1* and *pparγ* was not changed compared with their counterparts fed a control diet of maize silage, even though grass feeding significantly increased muscle ALA concentration.

Considering the Feedlot group as a control, the GPX1 and SOD2 enzyme activities were higher in Lucerne-fed animals and these differences in activity were reflected in the mRNA abundance. We have also seen in the present study that Lucerne feeding substantially increased muscle ALA whereas Feedlot diet substantially increased muscle LA concentrations. One thing we could draw from this is that diet-induced changes in muscle PUFA can alter expression and activity of SOD and(or) GPX antioxidant enzymes in the muscle tissue system. However, whether the altered activity of enzymes initiated the expression of genes or the altered expression of genes initiated the activity of enzymes was not clear, which requires further investigation. Animals and humans have a multi-dynamic defence mechanism regulated by enzymatic and non-enzymatic antioxidants in order to protect their tissues, organelles and individuals from oxidative stress and metabolic disorders [22,23,25]. The latter components have the capability to chelate, scavenge or decompose the damage causing hydroperoxides, transition metals, and other free radical forming substances or prooxidants and therefore protecting the body from adverse changes. Among them, glutathione peroxidase (GPX) and superoxide dismutase (SOD) have been reported as the major enzymatic antioxidants involved in defence mechanism in tissue systems of living organisms [21,22,23,40].

Two recent studies examined the activity and mRNA expressions of SOD and GPX antioxidant enzymes in mammary tissues in dairy cows with flax meal feeding at 5%–15% level or flax hull feeding at 10% or 500 g flax oil per day infused in the abomasum [32,41]. Those studies found that flax meal affected neither antioxidant enzyme activity nor mRNA expressions of SOD and GPX enzymes, but cows fed flax hulls, a rich source of lignans (secoislariciresinol diglucoside), had higher levels of *gpx1* and *sod1* mRNA expression and lower mRNA expressions of *gpx3*, *sod2* and *sod3* compared with cows fed nil flax hull diet. However, no effect of flax hull or oil on the activity of antioxidant enzymes was observed. Even though the fatty acid compositions of mammary tissues were not determined in both studies, it was [32] concluded that supplementing dairy cow diets with flax products high in omega-3 PUFA and lignans can modulate the activities of SOD and catalase enzymes that are responsible for the removal of free radicals leading to oxidative stress. Further, they [32] stated that the action of flax hull in altering the SOD activity and *sod1* mRNA abundance in the mammary tissue was related to the scavenging activities of polyphenolic compounds present in mammalian lignans that were converted from plant lignans of flax hulls added to the diet. Previous studies conducted in mice [42] and rats [43] showed that feeding fish oil rich in n-3 fatty acids of EPA and DHA enhanced GPX and SOD enzyme activities in spleen and liver tissues, respectively.

### 4.3. Relationships between Diet-Induced Increase in Muscle ALA and Gene Expression/Activity of Antioxidant Enzymes of GPX and SOD

There are no reports examining the relationships between the dietary enhancement of muscle ALA and activity of antioxidant enzymes of GPX and SOD or between the dietary enhancement of muscle ALA and gene expression of antioxidant enzymes of GPX and SOD. The current study showed that the GPX1 and SOD2 antioxidant enzyme activities linearly increased with the increase in ALA concentration in LL muscle of sheep, regardless of dietary treatments. At the same time, the significant increase in ALA concentration also linearly increased the mRNA expression in muscle LL, as observed by a reduction in delta CT. This diet-induced change in antioxidant enzyme activities and gene expressions may have beneficial effects on the performance and health and wellbeing of animals and humans.

The findings from recent animal studies support our statements: 1. animals grazing lucerne pasture or fed with flax flakes had better performance and carcass productivity than those fed other pasture diets or roughage with grain supplements [30,44,45]; 2. Biomarkers of blood oxidative stress measured as total antioxidant status were significantly higher with flaxmeal or flaxseed supplementation compared with oat grain supplemented groups [44] whereas isoprostanes concentration, an oxidative stress component was significantly lower with lucerne pasture feeding compared with feedlot diets [46]; 3. Sheep consuming lucerne (alfalfa) had a greater concentration of muscle vitamin E, a non-enzymatic antioxidant, than the grain-based feeding [28,46] whereas canola oil supplementation reduced blood oxidative stress substance of TBARS in goats [47]. Muscle tissues collected from sheep and goats from above-mentioned studies had higher concentrations of ALA and we believe this had some influence on animal performance and productivity through improved antioxidant potential, oxidative stress status and animal health. Taken together, these results demonstrate that diet-induced ALA resulted in altered activities and mRNA expression of antioxidant enzymes in muscle tissues, which, in turn, provided the animals with a higher antioxidant potential to alleviate oxidative stress and immune suppression, and protective mechanisms from adverse environmental conditions. However, further investigation is required to look at how different durations and concentrations of dietary interventions of feeding n-3 fatty acids affect health, welfare and productivity of lactating ewes and their lambs during hot and dry seasons, when availability and nutrient concentration of pasture (diets) is low, which can be applied to other species respectively.

## 5. Conclusions

Feedlot diet reduced ALA concentration by 50% in muscle compared with the Lucerne diet; whereas, Ryegrass and RyeFeedlot diets had 15% and 40% lower ALA concentration than Lucerne fed lambs, respectively. Muscle LA concentration was increased by 40%–45% in Feedlot animals compared to other treatments. Antioxidant enzymes activity of GPX1 and SOD2 in the muscle LL were significantly increased by the Lucerne diet compared with other dietary treatments. Gene expression of enzymes coding for *gpx1* and *sod2* in the muscle was greater for Lucerne fed lambs compared with Feedlot fed lambs. Muscle GPX1 and SOD2 enzyme activities were significantly linearly related to ALA. A positive linear relationship was observed between mRNA expression of *gpx1* or *sod2* and ALA concentration. We believe that the diet-induced increase in muscle ALA upstream the activities of GPX1 and SOD2, which in turn upregulate the expressions of GPX1 and SOD2 antioxidant enzymes in muscle tissues. This diet-induced change in activities and gene expressions of antioxidant enzymes may have beneficial effects on the performance, health and wellbeing of animals and humans, nevertheless, further research is warranted.

## Figures and Tables

**Table 1:** Dry matter (DM) content and nutritive characteristics of feedlot, lucerne pasture and annual ryegrass pasture used for feeding during the experimental period.

Items	Feedlot	Lucerne	Ryegrass
Dry matter, % as feed	91.1	32.3	32.4
Crude protein, % of DM	16.1	18.6	5.2
Metabolisable energy, MJ/kg DM	12.9	8.6	10.4
Acid detergent fibre, % of DM	9.0	33.5	26.3
Neutral detergent fibre, % of DM	19.6	44.2	45.5
Organic matter, % of DM	94.7	91.4	92.8
Dry matter digestibility, % of DM	82.7	59.6	69.6

**Table 2:** Primer sequences used for sheep specific real-time quantitative polymerase chain reaction amplifications utilized for determination of mRNA for target genes in muscle *longissimus lumborum* (LL) tissue.

Gene ^1^	Accession Number (Ovine)	Primer Sequence (5′ to 3′)
β-actin	NM_001009784.1	Forward: GTCCGTGACATCAAGGAGAAG Reverse: AGGAAGGAAGGCTGGAAGAG
*cpt1β*	NM_001009259.1	Forward: CCTGGAAGAAACGCCTGATT Reverse: CAGGGTTTGGCGAAAGAAGA
*gpx1*	XM_004018462	Forward: AGTTTGGGCATCAGGAAAAC Reverse: CCGAAGGAAGGCGAAGAG
*gpx4*	XM_004023249.1	Forward: GAGTTCGCTGCTGGCTATAA Reverse: CGGCAGATCCTTCTCTATCA
*nfκB*	EF_121765.1	Forward: ATTCAGCCCTTTGCCCATCT Reverse: ATGGGATGTCAGTGGCGTTA
*sod1*	NM_001145185.1	Forward: GGTTCCACGTCCATCAGTTT Reverse: CAATGGCAACACCATTTTTG
*sod2*	NM_001280703	Forward: TCACAGCATCTTCTGGACAA Reverse: TGCTCCTTATTGAAGCCAAG
*upc2*	NM_001280682.1	Forward: AAGGCCCACCTAATGACAGA Reverse: CCCAGGGCAGAGTTCATGT

^1^*cpt1β*, Carnitine palmitoyl transferase I β; *gpx1*, glutathione peroxidase 1; *gpx4*, glutathione peroxidase 4; *nfκB*, nuclear factor kappa B; *sod1*, superoxide dismutase 1; *sod2*, superoxide dismutase 2; *upc2*, Uncoupling protein 2.

**Table 3:** Individual fatty acid composition and total fat content (mg/100 g of muscle) of *longissimus lumborum* muscle from lambs consuming feedlot or grazing lucerne (alfalfa) pasture, ryegrass pasture or ryegrass pasture plus feedlot (RyeFeedlot).

	Feedlot	Lucerne	Ryegrass	RyeFeedlot	SED	*p*-Value
C10:0	4.8	5.8	5.0	4.2	0.68	0.13
C12:0	5.9	7.9	7.0	7.0	0.65	0.04
C14:0	86	102	93	90	9.1	0.27
C14:1	3.5	3.9	3.3	3.7	0.39	0.43
C15:0	8.8	11.3	10.6	9.7	0.64	0.01
C15:1	0.25	0.26	0.26	0.24	0.02	0.24
C16:0	566	607	577	499	42.5	0.09
C16:1	42.4	41.1	38.1	35.8	3.1	0.16
C17:0	27.7	29.7	29.4	25.3	2.1	0.07
C18:0	317	376	375	320	27.4	0.06
C18:1n-9cis	860	880	845	748	60.1	0.15
C18:2n-6 (LA)	139 ^b^	99 ^a^	93 ^a^	100 ^a^	5.4	0.001
*Cis*-9, *trans*-11 CLA ^1^	1.10	1.44	1.16	1.21	0.29	0.57
C18:3n-3 (ALA)	23.7 ^a^	50.7 ^d^	42.8 ^c^	30.8 ^b^	1.8	0.001
C20:4n-6 (AA)	34.6 ^b^	27.3 ^a^	28.0 ^a^	33.1 ^b^	1.3	0.001
C20:5n-3 (EPA)	12.8 ^a^	18.2 ^b^	17.3 ^b^	17.6 ^b^	0.74	0.001
C22:5n-3 (DPA)	12.9 ^a^	16.6 ^b^	15.9 ^b^	16.1 ^b^	0.58	0.001
C22:6n-3 (DHA)	3.7 ^a^	5.0 ^b^	4.8 ^b^	4.9 ^b^	0.32	0.001
Total n-3 fatty acids	53.4 ^a^	90.8 ^d^	81.0 ^c^	69.7 ^b^	2.43	0.001
Total n-6 fatty acids	180 ^c^	132 ^ab^	126 ^a^	139 ^b^	5.97	0.001
Ratio of n-6: n-3	3.5 ^c^	1.5 ^a^	1.6 ^a^	2.0 ^b^	0.15	0.001
Total fat (%)	2.2	2.3	2.2	2.0	0.14	0.13

^1^ CLA, conjugated linoleic acid = *cis*-9, *trans*-11 C18:2; LA, linoleic acid; ALA, alpha linolenic acid; AA, arachidonic acid; EPA, eicosapentaenoic acid; DPA, docosapentaenoic acid and DHA, docosahexaenoic acid. Total n-6 fatty acids include C18:3n-6, C20:3n-6, C20:4n-6 and C22:4n-6. Total n-3 fatty acids include C18:3n-3, C18:4n-3, C20:3n-3, C20:5n-3, C22:5n-3 and C22:6n-3. SED = Standard Error of Differences. Means with different superscripts a,b,c,d within a row are significantly different with each other.

**Table 4:** Statistical significance (*p*-values) for the relationships between muscle n-3 fatty acids and activities or gene expressions of glutathione peroxidase 1 (GPX1) and superoxide dismutase 2 (SOD2) antioxidant enzymes.

Response Variables	Terms					
	C18:3n-3 (ALA)			C20:5n-3 (EPA)		
	F-Statistic	DF	*p*-Value	F-Statistic	DF	*p*-Value
GPX1 activity	12.01	123.5	0.002	5.47	181.1	0.022
SOD2 activity	7.35	151.8	0.009	0.01	180.6	0.931
*gpx1* gene expression	6.75	121.8	0.017	0.11	164.4	0.744
*sod2* gene expression	3.05	123.2	0.094	0.20	163.4	0.653

ALA = Alpha linolenic acid; EPA = eicosapentaenoic acid; DF = Degrees of freedom.
